# Can vaccines control bacterial virulence and pathogenicity? *Bordetella pertussis*: the advantage of fitness over virulence

**DOI:** 10.1093/emph/eoac028

**Published:** 2022-08-03

**Authors:** Nicole Guiso, Benoit Soubeyrand, Denis Macina

**Affiliations:** Scientific Consulting, Paris, France; Blossom Vaccinology, Lyon, France; Sanofi, Vaccines Medical, Lyon, France

**Keywords:** vaccines, pathogenicity, *B. pertussis*, bacterial virulence, *C. diphtheriae*

## Abstract

Some vaccines, such as diphtheria toxoid and acellular pertussis vaccines (aPVs), may favor the emergence of less pathogenic strains of the respective bacteria they target. This review discusses the impact of the wide use of aPV on *Bordetella pertussis* phenotype evolutions and their beneficial consequences in the light of the diphtheria toxoid immunization program experience and structuring evidence review in a causal analysis following Bradford Hill’s causality criteria. All aPVs contain the pertussis toxin (PT), the main virulence factor of *B.pertussis*, alone or with one adhesin (filamentous hemagglutinin (FHA)), two adhesins (FHA and pertactin (PRN)) or four adhesins (FHA, PRN and two fimbriae (Fim 2/3)). In countries where the coverage of aPVs containing PRN is high, PRN negative *B.pertussis* isolates are increasing in prevalence, but isolates nonproducing the other antigens are rarely reported. We hypothesize that the selective pressure at play with PRN should exist against all aVP antigens, although detection biases may hinder its detection for other antigens, especially PT. PT being responsible for clinically frank cases of the disease, the opportunity to collect PT negative isolates is far lower than to collect PRN negative isolates which have a limited clinical impact. The replacement of the current *B.pertussis* by far less pathogenic isolates no longer producing the factors contained in aPVs should be expected as a consequence of the wide aPV use.

‘Nothing in biology makes sense except in the light of evolution’.

           Theodosius Dobjansky

## INTRODUCTION

Diphtheria and pertussis are among the oldest vaccine-preventable diseases. After a century of use of diphtheria toxoid and pertussis whole-cell vaccines (wPVs) and several decades of use of acellular pertussis vaccines (aPVs), what are the consequences on the agents of these diseases? Recent hypotheses about the evolutionary effects of vaccination suggest the imperfect nature of vaccines could increase or decrease the virulence of pathogens [1, 2]. Increased virulence may be true in animals for some pathogens in highly specific conditions (e.g. Marek disease) [3]. In humans, it seems more likely that the evolutionary pressure would result in the reduction of the pathogen virulence, at least for some bacterial vaccines. The replacement of toxicogenic *Corynebacterium diphtheriae* by nontoxicogenic strains in the population was the consequence of decades of toxoid immunization in countries with a high vaccination coverage. Could the same phenomenon be expected with *Bordetella pertussis* with the recent use of aPV, which are also toxoid-containing vaccines?

## DIPHTHERIA

The pathogenicity of *C.diphtheriae*, the agent of diphtheria is linked to virulence factors, including adhesion factors (pili and adhesins), agglutinins, O and K antigens and cord factor, which confers resistance to phagocytosis and facilitates tissue invasion, responsible for local symptoms at the site of infection (pharyngeal or cutaneous). The most important virulence factor of *C.diphtheriae* is the diphtheria toxin, an exotoxin that is responsible also for local symptoms as well as for the systemic symptomatology. Systemic absorption of diphtheria toxin results in toxic damage to organs such as the heart, kidneys and peripheral nerves [4].

The toxin is encoded by a highly conserved sequence of the tox gene of the ß-corynebacteriophage [5], which is repressed by a DNA-binding protein (DtxR) [4] when iron is available. In absence of iron in the vicinity, the diphtheria toxin expression is derepressed and inhibits protein synthesis in host cells leading to cell necrosis making the iron stored within host cells available to the bacteria. Of note, cases of frank diphtheria are far more contagious than cases of mild disease [6].

### Diphtheria vaccine

Diphtheria toxoid is prepared by treatment of the toxin with formalin that reduces its toxicity to clinically acceptable levels but retains its ability to elicit protective levels of antitoxin antibodies. Diphtheria toxoid–induced immunity is mediated by serum antitoxin IgG, directed mostly to the binding region of the toxin [4].

### Consequences of diphtheria vaccination

In countries with high vaccination coverage rates both for primary immunization and boosters in adolescents and adults, toxigenic *C.diphtheriae* strains have been replaced by nontoxigenic *C.diphtheriae* strains lacking or not expressing the tox gene [7, 8]. These strains can cause invasive infections such as endocarditis, septic arthritis and osteomyelitis, but are not the cause of diphtheria [9] BOX 1.

The most convincing evidence supporting this strain replacement as the epidemiological consequence of immunization with a toxoid is provided by the active follow-up of the national diphtheria immunization program in Romania implemented in 1958. Between 1958 and 1972, as more than 30 million doses of toxoid were administered, diphtheria cases and *C.diphtheriae* isolates were monitored. Over this 14-year period, the percentage of individuals immune to diphtheria increased from 60% to 97%, while the incidence of diphtheria cases fell from 600 cases to <1 case per 10 million inhabitants. At the start of the program, ∼90% of the *C.diphtheriae* analyzed were toxigenic. In 1972, more than 95% of the strains were nontoxigenic. It is striking that the virtual disappearance of toxigenic strains was deferred a few years after the disappearance of disease cases [10]. How can toxigenic isolates of *C.diphtheriae* disappear from the population following a vaccination which induces neutralizing antibodies against an exotoxin but without any effect on the bacteria itself?

In an unimmunized population, i.e. without (or low) anti-diphtheria toxin serum antibodies level, toxin appears to provide its *C.diphtheriae* with a competitive advantage over the existing *C.diphtheriae* flora which do not carry the tox gene, resulting in increased virulence, pathogenicity and transmission. In vaccinated hosts, mucosal antitoxin antibodies transform this competitive advantage into a competitive disadvantage. Indeed, the metabolism cost of producing proteins dedicated to iron release and its capture is extremely high for the bacteria, it could reach up to 75% of total protein synthesis [10]. This cost, which is no longer offset by an increase in iron resources when vaccine-elicited anti-toxin blocks the mechanism, becomes a negative selective pressure on toxigenic strains which are then gradually replaced by nontoxigenic strains. Nontoxigenic strains persist in the population, exposing it to outbreaks when the anti-toxin immunity of the population decreases and if a toxigenic strain is introduced within the population [11].

Diphtheria has vanished from areas with long-standing and thorough diphtheria toxoid vaccination programs, whereas the nontoxigenic isolates have persisted, a change that is attributable to the selective pressure exerted by the vaccine. Is a similar mechanism happening for pertussis with the recent implementation of aPVs?

## PERTUSSIS

Pertussis, often called whooping cough, is a highly infectious respiratory disease particularly dramatic for infants. The agents of the disease are gram-negative bacteria *B.pertussis* [12], more rarely *Bordetella parapertussis* [13]. *Bordetella pertussis* produces several adhesins such as the filamentous hemagglutinin (FHA), the fimbrial proteins (Fim2 and Fim3), the pertactin (PRN) and several toxins, one of the major ones being the pertussis toxin (PT) [14]. *Bordetella parapertussis* produces similar virulence factors except PT [15].

### Pertussis vaccines

The first pertussis vaccines developed were whole-cell vaccines (wPVs) containing inactivated bacterial suspensions of *B.pertussis*. The vaccine strains were of course collected during the pre-vaccine era and did not change thereafter. These pertussis vaccines were available since late 1940s in North America, late 1950s in Europe and since 1974 in the rest of the world, with the Expanded Programme on Immunization (EPI) [16]. The original strategy was a primary infant immunization followed, in some countries, by one booster. The EPI proved to be one of the most effective measures to reduce childhood morbidity and mortality from vaccine-preventable diseases [16]. However, after two or three decades of wPVs use, concerns arose regarding (i) adverse effects induced by these wPVs which, despite their being later proven to be reversible, led some countries such as Sweden or Japan to stop vaccination, (ii) low or declining initial effectiveness of some of the available wPVs, which also resulted in halted or decreased pertussis vaccination, (iii) waning of immunity after wPV vaccination and the change of the transmission pattern from a child-to-child transmission to an adult-to-infant transmission, which were pointed out first in the USA, then in France and in other European countries. This epidemiologic shift led to the realization of the importance of introducing vaccine boosters for older children, adolescents and adults. However, this was not possible with the wPVs due to the unacceptable reactogenicity beyond the age of 7. For all these reasons, aPV was then developed [16].

All aPVs contain PT, either alone or combined with one adhesin (FHA), two adhesins (FHA and PRN) or four adhesins (FHA, PRN and FIM-FIM3) [17]. These aPVs were proven to be efficacious and to induce fewer side effects in infants [18]. This led most high-income countries (HIC) to replace wPVs by the aPVs and to introduce vaccine boosters for older children. These vaccines are efficacious but, as with wPVs, the immunity they induce wanes over time. Therefore, adolescent and adult boosters were subsequently introduced in several HIC with aPV. Today the world is generally divided between low- and medium-income countries still using wPVs and a vaccine strategy with no or one booster, and HIC using aPVs and a vaccine strategy with several boosters.

### Impact of vaccination with aPV on the population of *B.pertussis* and other bordetellae?

*Bordetella pertussis* isolates not producing vaccine antigens circulated already during the prevaccine era and the wP era, but their prevalence was very low [19, 20]. As soon as 2007, the circulation of strains not producing PRN (PRN(−)) or not producing PT (PT(−)) was observed in France [20, 21]. Later on Japan described that PRN(−) strains were already collected at the end of 1990s, although Japan was the first country to use aPVs (with or without PRN) in the 1980s [22]. Similar strains, mostly PRN(−) but also PT(−) or FHA(−), were later on collected in other regions using aPVs such as Australia, USA, Japan and Europe [14, 23–29]. The recent analysis of the French collection from 1996 to 2018 confirmed the existence of the circulation of PT(−) and PRN(−) strains before the introduction of aPVs in France and the increase of the circulation of PRN(−) but also 3 PT(−), 3 FHA(−) and 8 Fim(−) isolates after the introduction of aPVs [30]. In 2007, in accordance with Soubeyrand and Plotkin hypothesis [2], one of us (N.G.) proposed that in HIC using aPVs and a vaccine strategy with several boosters, the vaccine coverage will increase and the prevalence of strains not producing vaccine antigens will also increase, as was observed with the impact of diphtheria vaccination [31, 32] BOX 2. We propose to evaluate the available evidence in the framework of the Bradford Hill’s criteria for causality to assess the plausibility of this hypothesized effect of aPV [33].

#### Strength of association

Very few studies have investigated the epidemiologic association between the use of aPV and the prevalence of strains of *B.pertussis* not expressing aPV antigens, and for good reasons. First and foremost, the transition to aPV is largely historical and occurred generally at a large-scale replacement, providing little opportunity to evaluate the phenomenon. However, more importantly, the detection of vaccine antigen loss is likely biased since strains tend to originate from cases of symptomatic infection, more likely caused by strains not lacking most vaccine antigens. One notable exception is a study conducted on PRN(−) strains in the US pertussis surveillance network, which showed that pertussis cases >1 year of age who were age-appropriately aPV vaccinated had 2.7-fold (95% confidence interval 1.2–6.1) higher odds of being infected by a PRN(−) strain than completely unvaccinated cases [34]. This finding was interpreted as evidence of the selective pressure aPV vaccines may be eliciting in selecting PRN(−) strains with a better fit for spreading in highly vaccinated populations BOX 3.

#### Consistency and specificity of observation

Despite the inherent detection biases in observing the evolution of circulating *B.pertussis* strains, and to surveillance capacity differences between aPV and wPV using countries, there are indications from available evidence that the shift in circulating strains from vaccine antigen producing to not producing is consistently and specifically associated with the use of aPV. Firstly, the analysis of strains genotypes changes over time has shown in multiple aPV using countries that the shift increased in magnitude in years following the transition to aPV [20, 24, 28, 30]. On the contrary, no or very few PRN(−) or PT(−) or FHA(−) isolates are collected in regions where wPV is currently used such in Argentina, Brazil or Tunisia [35–37]. Interestingly, changes have also been observed in France since 2007 in the population of *B.parapertussis*. This species which produces similar antigens (such as PRN, FHA, Fim) as *B.pertussis* presents a higher prevalence of strain no longer producing PRN [14, 15] indicating the population-level immunity elicited by aPV also affects *B.parapertussis*. France is one of the only countries reporting such an evolution of this other closely related *Bordetella* species, and this observation is quite puzzling since contradictory results have been published regarding the possible impact of aPV-induced immunity on the protection against this species [15]. However, the prevalence of this *Bordetella* species is not increasing since the introduction of aPV suggesting that the immunity induced by these aPV protects against this species and has a possible impact on their evolution similar to that on *B.pertussis*.

More broadly, one can also wonder about the potential impact of the introduction of aPVs containing a limited number of *B.pertussis* antigens (PT, PRN, FHA, FIM 2 and 3) in replacement of the wPVs, which contain all the antigens of the bacterium, on another emergent *Bordetella* species, *Bordetella holmesii* through negative selection pressure instead of positive selection pressure. Recently, this *Bordetella* species has been increasingly isolated from respiratory specimens obtained from otherwise healthy persons with pertussis-like illness [38]. It is not known whether the bacterium causes a pertussis-like illness, or it is only incidentally detected in patients infected by other pathogens. In fact, it is frequently misidentified as being the cause of whooping cough along with *B.pertussis*, because routine polymerase chain reaction (PCR) diagnostic tests for pertussis are not species-specific [38]. Excluding a gene encoding a protein related to FHA, genes encoding adhesins and toxins implicated in the *Bordetella* pathogenesis, and which are contained in aPVs have not been detected in *B.holmesii* isolates. Therefore, aPVs cannot *a priori*, exert a positive selection pressure on *B.holmesii* because the bacterium does not produce the vaccine antigens (PT, PRN, FHA, FIM 2 and 3) apart from an ‘FHA –like’ [38]. Epidemiological studies would be extremely useful to improve our current epidemiological understanding and for addressing the many yet unanswered questions about *B.holmesii*.

#### Temporality of effect

The few available data from historical isolates do show that *B.pertussis* strains not expressing vaccine antigens pre-existed vaccines altogether and persisted during the wPV period [19, 20, 39]. Nevertheless, increases in their prevalence have been documented by several studies as occurring clearly after the switch to aPV. This was found in France but also in other European countries, in Japan, the USA, Canada and Australia [24, 26, 28–30, 40]. These increases, or at least their documentation, were generally delayed by several years after the aPV was adopted [24, 28, 29], which may question whether the aPV is truly causal in the predominance of their circulation. However, the effect must be considered in the broader epidemiologic context. The aPV were adopted first only for pediatric vaccination, limiting their effect and leaving large portions of the population with immunity either derived from wPV or from natural infection, or not immune. As a result, the selective effect of aPV could only really take hold once the immunity they induce increases in the prevalence and replaces wPV immunity or natural infection immunity. In any case, the observation that the transition of aPV preceded the increase in prevalence of *B.pertussis* strains not expressing vaccine antigens fulfills the temporality criterion.

#### Biological gradient

This criterion cannot be applied *stricto sensu* in this evaluation given the fact that aPV have a generally consistent dose composition across vaccines. Nevertheless, one may consider the effect of time elapsed since aPV adoption as a form of ‘dose escalation’ in the effect we are evaluating here. In fact, as time progressed from the switch from wPV to aPV, increasing proportions of the population have anti-pertussis immunity defined by aPV antigens, escalating progressively the selective pressure on vaccine antigen expression. In addition, one may consider the transition from wPV to aPV as a form of dose escalation in relation to antigen content. Indeed, in the aPV, the proportion of each antigen relative to the total amount of antigens is very much increased compared to the proportion they represent in wPV relative to the thousands of other antigens present in the bacterium. This would be expected to translate in a more focused and stronger immune response against these specific antigens after aPV vaccination compared to wPV vaccination. On this basis, it is interesting to note that an analysis across nine European countries with diverse timing of the transition to aPV showed that the longer the period since the introduction of aPVs containing PRN, the higher the frequency of circulating PRN(−) isolates [28]. Similarly, the transition from wPV to aPV may affect the selective pressure on PT production. In fact, aPV vaccines are formulated to contain higher amounts of PT than wPV in which this antigen is not quantified for formulation. The rarity of detection of PT(−) isolates makes demonstrating this hypothesis difficult, but it is interesting to note that among the few cases reported in literature, the only two identified during the wPV period in France were completely unvaccinated, while the ones reported in aPV context were unvaccinated for one and vaccinated with 1 or 3 aPV doses for the others [21, 25, 30].

#### Biological plausibility and coherence with previous knowledge

Whether it is in humans or animals, several studies have generated evidence pointing to the fact that the loss of PRN provided *B.pertussis* with a better fit in infecting especially vaccinated individuals [34, 41, 42]. It was actually recently hypothesized based on human and animal studies that PRN is the sole antigen in aPV that generates complement-mediated bactericidal antibodies suggesting that aPV might be a driver of the loss of PRN [43]. In fact, Ma *et al.* [44] highlighted the three aspects of PRN for selective loss such as its functional redundancy, the relatively longer functional persistence of antibodies against it, and its close location to the surface membrane for productive complement fixation. However, as they mentioned ‘sufficient sustained selective pressure against any particular antigen is likely to lead to its loss or change. Will the loss of these factors result in strains attenuated in virulence such that they become more like commensals than pathogens? Or is PRN really the only antigen that can be lost without serious fitness costs?’. As described in the studies cited above there are now several arguments suggesting that the immunity induced by aPV has a role in the increase of the prevalence of PRN(−) strains. However, this immunity might also have a role on other antigens such as PT or FHA or Fim, although their prevalence is considered anecdotal. The five subunits of PT (Ptx1–Ptx5) are highly conserved according to their sequence, predicted structure and antigenicity and are under purifying selection whereas PRN and FHA are under statistically significant diversifying selection [45]. However, there are more than 200 insertion sequences on the chromosome of *B.pertussis* that can induce the deletion of the five PTx genes as observed previously [21]. In fact, according to Etskovitz *et al*. [45] aPV-derived immunity does not impact PT but may apply evolutionary pressure to PRN and FHA to undergo diversifying selection. According to Zeddeman *et al.* [27] using a murine model of infection bacterial populations isolated from the lungs shifted to an FHA(−) phenotype. Loss of FHA expression was strongly selected for in PRN(−) strains in the lungs following aPV but not wPV vaccination.

Because samples are taken from individuals who experience whooping cough, and that PRN has only a limited impact on the clinical symptomatology, PRN(−) isolates can be as easily isolated as PRN(+) ones. Because PT is the main virulence factor of *B.pertussis* and responsible for frank cases of the disease, the opportunity to collect PT(−) *B.pertussis* isolates is far lower than to collect PRN(−) isolates [21, 25]. It might be the same for FHA(−) strains. During the Pertinent study, a European hospital-based study, some patients were biologically confirmed by PCR but did not have symptoms, suggesting that they might have been infected by a PT(−) strain [46]. It will then be of interest to follow the clinical symptoms of patients with a positive pertussis PCR, including coinfections, according to their age, vaccination status and contacts. If the clinical symptoms are typical, it could be an infection with PRN(+) or PRN(−) isolates but if the symptoms are milder, it could be a PT(−). In particular, it might be important to know whether an infant with a positive PCR had an hyperlymphocytosis.

#### Experimental evidence

Considering the dynamics involved in bacterial population changes and their association with human immunity and epidemiologic changes, it is difficult to conceive true human experiments that could illustrate the impact of aPV on the expression of vaccine antigens in *B.pertussis* populations. This said, one particular analysis in Japan indicated that, while the frequency of PRN(−) isolates detection had been increasing in this country since the 2000s with the use of PRN-containing aPV, the frequency of PRN(−) isolates detection decreased, in particular after 2012, with the replacement of aPV vaccines with formulations that do not contain PRN [47]. This observation of reversal of the effect with the removal of PRN-containing aPV may be interpreted as quasi-experimental evidence of the effect of aPV on the phenotype of *B.pertussis* populations.

#### Analogy

By exerting selective pressure on microbes, population immunity, whether acquired through natural exposure which goes back to the dawn of time or artificial (vaccine) more recently, forces them to evolve. The clearest analogy to our hypothesis was discussed at the beginning of this article with the example of the impact of diphtheria toxoid vaccines on the toxigenic phenotype of circulating *C.diphtheriae* populations.

## CONCLUSION

In all areas with long-standing diphtheria toxoid vaccination programs, diphtheria is controlled. Nontoxigenic *C.diphtheriae* strains now circulate with a prevalence, which is difficult to estimate in absence of epidemiological studies in the general population. The spread of these nontoxigenic strains is attributed to selective pressure induced by the vaccine after immunizing children as well as adolescents and adults. Our prediction is that in HIC, with increasing aPV coverage, not only in infants but also in children, adolescents and adults, the prevalence of strains not producing vaccine antigens might be higher than it is presently described due to a detection bias. Sequencing the DNA of *B.pertussis* positive samples by PCR to know whether mutations in the genes encoding PRN, FHA, PT or Fim in correlation with clinical symptoms of the patients might give the answer. Despite the limitations posed by detection biases, the evidence available today points to a similar mechanism at play in the interaction between *B.pertussis* and aPV, with positive indications that Bradford Hill’s criteria for causality are fulfilled to support the selective pressure exercised by aPV-elicited immunity on the expression of aPV antigens by circulating *B.pertussis* strains BOX 4. The wide use of aPVs in all age groups might lead to the same mechanism of selection pressure as seen for diphtheria, leading to the progressive replacement of the current *B.pertussis* by far less pathogenic *B.pertussis* no longer expressing the virulence factors PT, FHA, PRN and/or Fim contained in aPV. PRN(−) *B.pertussis* strains were shown to be almost as pathogenic as those producing PRN in animal models contrary to PT(−) isolates [21, 41, 42] despite a higher fitness than PRN(+) isolates. They were also shown to be almost as pathogenic in infants [34, 48, 49]. It has been hypothesized that the increase of the prevalence of PRN(−) isolates might decrease the aPV efficacy [45, 50]. The model developed by Jayasundara *et al.* [50] suggests that the emergence of PRN(−) strains in the recent epidemics in Australia is vaccine-driven and is the cause of the increase in the incidence of the disease. However, the decrease in disease severity among vaccinated individuals is still apparent [48] and vaccination against pertussis remains an important public health measure since aPV effectiveness did not seem to be reduced in the context of high prevalence of PRN(−) strains in the USA [51]. If our assumption is correct, then it can be expected that the remaining nonvaccinated persons will be indirectly protected against the risk of severe whooping cough. Running pertussis epidemiology studies in general population looking at the production of virulence factors, especially PT, could confirm our hypothesis and will help to improve the pertussis disease management thanks to better adapted aP immunization programs.

**Conflict of interest:** N.G. and B.S. are independent consultants in vaccinology and report no conflict of interest related to the topic addressed in this publication. D.M. is an employee of Sanofi and holds shares of the Sanofi group.

BOX 1 Diphtheria toxoid vaccines impact on circulating pathogen

In all areas with long-standing diphtheria toxoid vaccination programs, diphtheria is controlled. Nontoxigenic *C.diphtheriae* isolates now circulate. The spread of these isolates is attributed to selective pressure induced by the vaccine after immunizing children as well as adolescents and adults.

BOX 2 Acellular pertussis vaccine hypothesis

Several decades of the wide use of aPVs in all age groups might lead to the same mechanism of selection pressure leading to the progressive replacement of the current *B.pertussis* isolates by far less pathogenic isolates no longer expressing the virulence factors PT, FHA, PRN and/or Fim contained in aPVs.

BOX 3 The pertactin example

It is now well established that in countries using aPVs containing PRN, with a high vaccine coverage, an increase in the circulation of PRN(−) isolates is observed. The circulation of isolates nonproducing other vaccine antigens which is currently considered insignificant might actually reflect an observational bias due to an absence of detection of those isolates.

BOX 4 The pertussis toxin loss hypothesis

It is well established that PRN(−) isolates are almost as virulent as PRN(+) isolates, and they can be collected as easily as PRN(+) isolates. We hypothesize that because PT is the main virulence factor of *B.pertussis* and responsible for frank cases of the disease, the opportunity to collect *B.pertussis* PT(−) isolates is far lower than to collect PRN(−) isolates, explaining that the phenomenon is wrongly considered marginal. Today, most epidemiological data rely on pertussis PCR based on insertion sequence elements which therefore do not distinguish between isolates producing vaccine antigens or not. A wide epidemiological study based on sequencing the DNA contained in samples of positive PCR of subjects with mild clinical symptoms of whooping cough could confirm our hypothesis.
